# Conjugated Polymer Modifying TiO_2_ Performance for Visible-Light Photodegradation of Organics

**DOI:** 10.3390/polym15132805

**Published:** 2023-06-24

**Authors:** Cristina Giorgiana Coromelci, Elvira Turcu, Florica Doroftei, Mircea Nicolae Palamaru, Maria Ignat

**Affiliations:** 1 Faculty of Chemistry, “Alexandru Ioan Cuza” University of Iaşi, 11 Carol I Blvd, 700506 Iaşi, Romania; cristina.coromelci@uaic.ro (C.G.C.); palamaru@uaic.ro (M.N.P.); 2 Institute of Interdisciplinary Research, Department of Exact Sciences and Natural Sciences, “Alexandru Ioan Cuza” University, 11 Carol I Blvd, 700506 Iasi, Romania; 3 Department of Inorganic Polymers, “Petru Poni” Institute of Macromolecular Chemistry, 41A Grigore GhicaVoda Alley, 700487 Iasi, Romania; mahu.elvira@icmpp.ro (E.T.); florica.doroftei@icmpp.ro (F.D.)

**Keywords:** conjugated polymers, TiO_2_, mesoporosity, visible light, organic pollutants

## Abstract

Up to now, the use of TiO_2_ has been considered a promising advanced technology for organic pollutants removal from air or water, since it has high biological and chemical stability, high photoactivity, low toxicity, and low-cost production. However, there are issues to be addressed in enhancing TiO_2_ performance, and one of the current key issues is redesigning UV-active photocatalysts and making them active in the visible region of the electromagnetic spectrum. This way, solar light absorption will be insured, and thus, a more efficient photocatalyst could be obtained. For this reason, conjugated polymers and their derivatives are considered to act as photosensitizers, being able to shift the TiO_2_ activity from the UV to the visible region. Therefore, this study focuses on the synthesis of TiO_2_/conjugated polymer systems, which was accomplished by the deposition of poly-3,4-ethylene-dioxy-thiophene (PEDOT [-C_6_H_4_O_2_S-]_n_), a low-band semiconductor with an excellent stability due to its extending π-conjugated electron system, on titania nanoarchitecture. First of all, a TiO_2_ nanoarchitecture was synthesized by an ultrasound-assisted sol–gel method. Then, TiO_2_/PEDOT systems were obtained and characterized by using different techniques such as X-ray diffraction, Fourier Transform Infrared Spectroscopy, Scanning Electron Microscopy, UV–Vis diffuse reflectance, and N_2_ sorption measurements. The synthesized composites confirmed their mesoporosity and lower band gap values compared to bare titania, which clearly shows the ability to work as photocatalysts under visible-light activity. Further, we demonstrated that an organic pollutant, Congo Red dye, used as a model molecule could be photodegraded with the synthesized TiO_2_/PEDOT systems, with efficiencies of up to 95% in the case of T_conv_PEDOT under UV light and up to 99% for T_conv_PEDOT under visible-light irradiation, accomplishing in this way a successful synthesis of visible-light-activated titania photocatalyst.

## 1. Introduction

Overexploitation and pollution, alongside climate change, have created a major challenge in assuring the necessary quantity of drinking water around the globe [1]. Accordingly, researchers around the world are looking for feasible methods of wastewater cleaning to return wastewater for reuse in different industrial processes or agriculture activities [2]. Over the past decades, semiconductor photocatalysis, considered an affordable and uncomplicated technology for wastewater treatment, has attracted considerable attention. Thus, extensive research has been conducted on many nanostructured metal-oxide semiconductors such as TiO_2_, ZnO, and WO_3_ [3]. Despite being one of the most commonly employed photocatalytic materials [4], TiO_2_ has a relatively wide band gap ranging from 3.0 to 3.3 eV [5,6]. As a result, bare TiO_2_ exhibits photoactivity only under UV excitation, making it disadvantaged from this point of view. As is well known, at the Earth’s surface, UV light is about ∼3–4% of the solar spectrum, that being one reason for researchers to explore and develop TiO_2_-based photocatalyst systems with an improved visible-light response. Concerning this aspect, a scientific solution is proposed focusing on the achievement of a fast photoinduced charge separation and a slow charge recombination by photogenerated electron acceptors from the photocatalytic TiO_2_ nanoparticle surface [7]. Such an effect has been obtained by coupling semiconducting polymers with TiO_2_ photocatalyst, improving TiO_2_ photocatalytic efficiency [8]. However, conjugated polymers and their derivatives can act as photosensitizers and have shown excellent stability due to their extending π-conjugated electron systems. These polymers demonstrate robust light absorption capabilities across a broad spectrum, encompassing the ultraviolet, visible, and near-infrared (NIR) regions, due to which they can serve as efficient photosensitizers for TiO_2_ [3].

Thus, according to Saianand et al. [9], the heterojunction occurring between semiconducting polymers, such as polyaniline (PANI) and polypyrrole (PPy), and TiO_2_ photocatalyst led to materials with improved properties regarding visible-light absorption, photogenerated charge separation, and material stability. Likewise, Sangareswari and Sundaram [10] tested PPy-TiO_2_ nanocomposite prepared by the chemical oxidative polymerization method for methylene blue removal from aqueous systems, with a removal efficiency of up to 93%. Moreover, Dimitrijevic et al. [11] synthesized TiO_2_/polypyrrole nanocomposites to be used as visible-light-activated photocatalysts for methylene blue degradation and H_2_ production from water.

Besides polyaniline (PANI) and polypyrrole (PPy), recent studies reported that polythiophene and its derivatives exhibit photocatalytic activity [12] and could improve the visible-light response of other photocatalysts. Thereby, due to its high stability in the presence of oxidative species, such as hydroxyl radicals, poly-3,4-ethylene-dioxy-thiophene (PEDOT) has demonstrated a synergic effect in PEDOT-based nanocomposites used in the photodegradation process of methylene blue dye [13]. In addition, Dagar et al. [14] demonstrated the efficiency of a PEDOT/NiO/Fly ash cenosphere composite in the photodegradation process of methyl orange under visible-light irradiation, while Abdiryim et al. [15] studied the photocatalytic activities of PEDOT/ZnO composites, achieving a degradation efficiency of methylene blue of up to 96.6% under natural sunlight as a source. As well, a hybrid film consisting of TiO_2_-PEDOT:PSS (poly(3,4-ethylenedioxythiophene:polystyrene sulfonate)) heterojunction photocatalyst has been investigated by Vavilapalli et al. [16], demonstrating photocatalytic efficiencies of methylene blue and bisphenol-A removal, under simulated sunlight, of about 99% and 93%, respectively.

To the best of our knowledge, very few studies reporting the preparation and photocatalytic activity investigation of dual TiO_2_/PEDOT systems are available. For example, Katančić et al. [3] studied the photocatalytic activity of TiO_2_ P25–PEDOT systems in the removal process of bisphenol A, under simulated solar irradiation, prepared by using ammonium persulfate and iron (III) chloride as oxidants, concluding that the prepared photocatalyst is more effective for bisphenol A removal under solar irradiation than TiO_2_ P25 material. Furthermore, Liu et al. [17] infused TiO_2_ nanofibers with PEDOT and proved that the synthesized photocatalyst has a higher initial degradation rate constant than its non-PEDOT-infused counterparts in the degradation process of phenazopyridine.

Therefore, the literature survey guided us to find extended applications of developed porous titania materials, developed in our laboratory [18] using an ultrasound-assisted synthesis procedure that led to a TiO_2_ material with structural, morphological, and textural features greater than TiO_2_ P25. Thus, this study describes the preparation of TiO_2_/conjugated polymer systems by depositing poly-3,4-ethylene-dioxy-thiophene (PEDOT) over TiO_2_, considering iron (III) chloride as oxidant, and explores the photocatalytic potential of the newly synthesized composites in the photodegradation of Congo Red dye, under UV- and visible-light irradiation, comparatively.

## 2. Materials and Methods

### 2.1. Materials

Pluronic^®^ F-127 [(C_3_H_6_O·C_2_H_4_O)_x_, mol wt ~12,600 g/mol], titanium (IV) isopropoxide [Ti(OCH(CH_3_)_2_)_4_], isopropyl alcohol [C_3_H_8_O] (IPA), iron (III) chloride [FeCl_3_·6H_2_O], 3,4-Ethylendioxythiophen [C_6_H_6_O_2_S] (density ρ = 1.331 g/mL at 25 °C), Congo Red [C_32_H_22_N_6_Na_2_O_6_S_2_] dye, potassium chromate [K_2_CrO_4_], 1,4-hydroquinone (H_2_Q), and disodium ethylenediamine tetra-acetate (CIII) were used as purchased from Sigma–Aldrich, without further purification. All used chemicals were analytical grade. In all experiments, ultrapure water prepared with an Elga PURELAB Option-R7/15 system was used.

### 2.2. Synthesis of Mesoporous TiO_2_

Ultrasound-assisted template sol–gel technique was employed in the synthesis procedure of mesoporous TiO_2_ material. Thus, in a typical synthesis, 10 g of pluronic^®^ F127 was dissolved in water/ethanol mixture (50/50 *v*/*v*) to allow micellization to occur. Then, 18 mL of titanium isopropoxide was added dropwise to the obtained mixture, and an ultrasound treatment was applied. Thus, an acoustic horn-probe tip (Vibra Cell, 750 W) was immersed in the reaction mixture and sonicated for 1 h, with a 3 on/1 off pulsation mode. The yellow milky suspension thus obtained was separated by centrifugation, and the resulting solid was then washed several times with ultrapure water and dried overnight at 60 °C. Additionally, the calcination process was carried out using 2 different methods: first, in a conventional furnace (Carbolite ELF 11/6) at 450 °C for 4 h with a heating rate of 1 °C per minute, and second, in a multimode microwave (MW) oven at 700 W power for 30 min. The prepared samples were labeled as T_conv_—calcined in a muffle furnace, and T_mw_—calcined in an MW oven, respectively.

### 2.3. Synthesis of Mesoporous TiO_2_/PEDOT Systems

To obtain post-synthesis TiO_2_/PEDOT systems, a mixture containing the synthesized titania (T_conv_ and T_mw_, respectively), iron (III) chloride (FeCl_3_·6H_2_O, 1M), and EDOT monomer, in the molar ratio TiO_2_:FeCl_3_:EDOT = 1:1.6:0.4, was vigorously stirred for 24 h, at room temperature, allowing PEDOT to be polymerized between TiO_2_ nanoparticles. Afterward, the solid composite materials were separated by filtration. Then, the resulting samples were dried overnight at 60 °C and denoted as T_conv_PEDOT and T_mw_PEDOT, respectively. For comparison, the same procedure without the addition of TiO_2_ was carried out to prepare a PEDOT sample.

### 2.4. Characterization of the TiO_2_/PEDOT Systems

The structural, textural, and morphological characteristics of the synthesized systems were studied by X-ray diffraction, by the nitrogen sorption technique, and by Scanning Electron Microscopy (SEM), respectively. The surface chemistry of the samples was studied by Fourier Transform Infrared Spectroscopy (FTIR) and X-ray Photoelectron Spectroscopy (XPS). The optical properties of the prepared powders were highlighted by representing the Tauc plots derived from the registered UV–Vis diffuse reflectance spectra.

Further, a range of laboratory equipment was employed: for the XRD pattern recording, a Shimadzu LabX XRD-6000 (Kyoto, Japan) advanced diffractometer was employed for the analysis. The instrument utilized Cu–K radiation with a wavelength (λ) of 1.5406 Å and operated in continuous mode, in the range of 20–70° (2-theta). The data obtained allowed us to calculate the crystallite sizes using Scherrer′s equation:

(1) $$D_{Scherrer}=K*\lambda /\left(\beta *cos\theta \right)$$

where $D_{Scherrer}$ represents the average size of the TiO_2_ crystallite, while *K* is the shape factor, a dimensionless parameter with a value typically close to unity (*K* = 0.89); *λ* denotes the used X-ray wavelength; *β* refers to the extent of broadening at half of the maximum intensity (FWHM), measured in radians; and *θ* represents the Bragg angle. Similarly, the Williamson–Hall (W-H) theory was applied to XRD data to determine the crystallite size as well, $D_{W-H}$, and the strain (*ε*).

A NOVA 2200e instrument (Quantachrome Instruments, Boynton Beach, FL, USA). was used for the N_2_ sorption investigation exploring the textural parameters, such as BET specific surface area, determined by applying the Brunauer–Emmett–Teller (BET) theory; the pore diameter, estimated by considering density functional theory (DFT); and the total pore volume, calculated at the relative pressure of 0.95. A Quanta 200 Scanning Electron Microscope (FEI Company, Hillsboro, Oregon, United States) was used to capture SEM images. The FTIR spectra were acquired on a Bruker Vertex spectrometer at a spectral resolution of 2 cm^−1^, in the wavenumber region of 4000–400 cm^−1^, using KBr pellets. For the registration of XPS spectra, a PHI 5000 VersaProbe photoelectron spectrometer manufactured by Ulvac-PHI, Inc. (Chikasaki, Japan) was utilized. The instrument employed monochromated Al Kα radiation with an energy of 1486.7 eV. UV–Vis DR spectra were registered on a Shimadzu UV-2450 spectrophotometer equipped with an integrating sphere, using an MgO sample as a reference, to obtain insight into the indirect band gap energy values. In this regard, the Kubelka-Munk function (Equation (1)) was applied, and further, the band gap was calculated by applying Tauc theory for indirect allowed transitions and plotting (F(R∞)hυ)12=f(hυ)) graphs.

(2) $$F\left(R\right)=\frac{{\left(1-R\right)}^{2}}{2R}=\frac{\alpha }{s}=\frac{A*c}{s}$$

where *R* is reflectance, *α* is absorption coefficient, *s* is scattering coefficient, *c* is concentration of absorbing species, and *A* is absorbance.

### 2.5. Investigation of Photocatalytic Activities of TiO_2_/PEDOT Composites

The prepared samples showed photocatalytic activity in the process of degradation of Congo Red (CR) dye. Thus, aqueous solutions, with initial concentrations of 50 and 100 mg/L CR, were considered in photocatalytic experiments. As well, catalyst dose in the photocatalytic experiment varied, and different catalyst:dye weight ratios (of 4:1, 1:1, and 1:2) were considered to find out the optimal conditions for reaching the highest photodegradation activity. In all photocatalytic experiments, a volume of 50 mL of CR solution was taken for treatment. First, the suspensions of prepared TiO_2_/PEDOT composites in CR aqueous solution were magnetically stirred in the dark for 30 min to reach the adsorption–desorption equilibrium of CR molecules on the catalyst surface. Therefore, the zero-time reading corresponds to the instant when the lamp was switched on. A 15 W Hirolab UV Hand Lamp ($\lambda_{1}=254\;\mathrm{nm}$, 0.153 mW/cm^2^ power density and $\lambda_{2}=365\;\mathrm{nm}$, 0.312 mW/cm^2^ power density) and a 400 W sodium vapor lamp (λ = 589 nm, and 14.8 mW/cm^2^ power density) were used during the UV- and the visible-light-activated experiments, respectively. The lamps’ power density measurements were conducted at the same distance as the samples were placed, using an HD2102.2 Delta Ohm photo-radiometer equipped with a SICRAM detection module. In the photocatalytic experiments, samples of 5 *mL* were extracted from the suspension at predetermined time intervals and were promptly separated through 45 µm PTFE Isolab syringe filters. The filtered samples were then subjected to analysis by measuring the absorbance at the wavelength of 496 nm, on a Shimadzu UV-2450 spectrophotometer, characteristic of the CR dye maximum absorbance. Total organic carbon in the liquid samples was determined using a multi N/C 3100 TOC Analyzer (Analytik Jena AG, Jena, Germany) equipped with a chemiluminescence detector (CLD). Different scavengers were used in order to find out which are the reactive species responsible for photocatalytic degradation: 10 mmol L^−^^1^ IPA, 0.25 mmol L^−^^1^ H_2_Q, 25 mmol L^−^^1^ K_2_CrO_4_, and 10 mmol L^−^^1^ CIII.

## 3. Results and Discussion

### 3.1. Characterization of the Synthesized Titania Samples

#### 3.1.1. XRD Measurements

**Figure 1:**
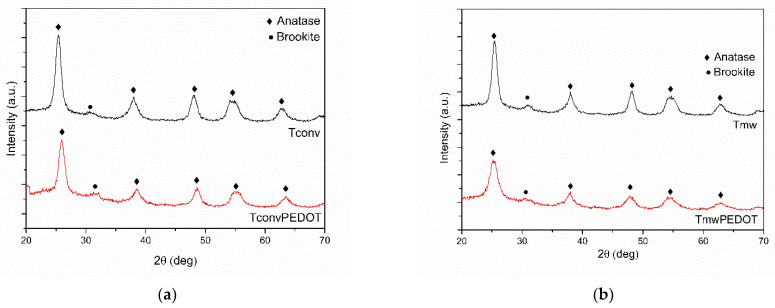
X-ray diffraction patterns for prepared TiO_2_ samples: (**a**) T_conv_ and T_conv_PEDOT; (**b**) T_mw_ and T_mw_PEDOT.

The structural properties of TiO_2_/PEDOT composites were investigated using X-ray analysis. Therefore, the registered diffractograms were plotted and are shown in Figure 1. The found well-defined diffraction peaks indicate the presence of crystalline anatase phase in the synthesized samples. Thus, as depicted in Figure 1, characteristic peaks associated with the planes in the crystal lattice of the tetragonal anatase TiO_2_ phase, namely (101), (103), (200), (211), and (213), corresponding to the 2θ values of 25.38, 38, 48.06, 54.54, and 62.52 degrees [19], are observed in all registered X-ray diffraction patterns. Moreover, the orthorhombic brookite (211) [20] plane is also noticed at 2θ = 30.8 degrees, suggesting a mixture of both titania phases. Regarding the PEDOT-containing samples, the (020) reflection characterizing π−π stacking of the polymeric network [21] found at 26.5° could be the reason for the intensity decrease in the main (101) peak.

Further, Williamson–Hall (W-H) theory, which involves the use of data from all the XRD peaks, was also applied to determine the crystallite sizes (D_WH_) and strains (ε), the results of which are presented together in Table 1.

Thus, the calculations for the crystallite diameters, using the Scherrer formula, were facilitated by the well-individualized characteristic peaks at 2θ = 25.34 and 2θ = 26.5 deg, for pure anatase and PEDOT samples, respectively [19]. As observed in Figure 1, the anatase characteristic diffraction peaks widen as PEDOT is loaded on the TiO_2_ matrix, indicating the polymer decoration results in the individualization of smaller crystallites compared with the pure TiO_2_ samples (Table 1). This finding could be useful for heterogeneous photocatalysis, due to the increased interface contact of the photocatalyst with the reactant molecule [7]. The deposition of the black conjugated polymer on the white TiO_2_ nanoparticles is demonstrated also by the color change to bright gray. However, when the conjugated polymer has been attached to the TiO_2_ surface, a slow decrease in diffraction peak intensity (101) is observed, which could be attributed to the crystal face inhibition effect, as has been reported in the literature elsewhere [22]. The decrease could also be associated with the amorphous phase of the in situ polymerized PEDOT [23]. An interesting finding is deduced from XRD data regarding the evolution of the crystallite size during the PEDOT deposition process on the T_mw_ and T_conv_ samples. Therefore, a higher decrease of 62% in the crystallite size was found for the T_mw_PEDOT system, while for the T_conv_PEDOT, the decrease is only 7%. This finding is in good agreement with the Williamson–Hall theory calculations of crystallite size, which reveal the same tendency. This could be explained by the formation of bulk amorphous PEDOT regions [24,25] within the TiO_2_ structure that enlarge the peak width with a decrease in the peak’s intensity, these parameters being directly responsible for the crystallite size calculations.

#### 3.1.2. N_2_ Sorption Measurements

**Figure 2:**
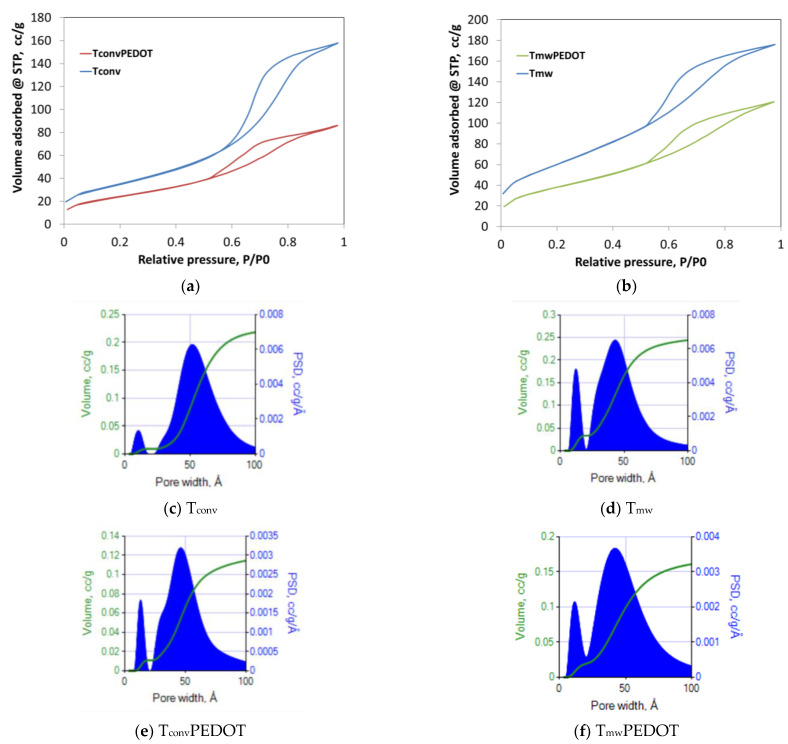
Textural characterization of TiO_2_/PEDOT composites: N_2_ adsorption/desorption isotherms for T_conv_ and T_conv_PEDOT (**a**) and for T_mw_ and T_mw_PEDOT (**b**); DFT pore size distributions for T_conv_ (**c**), T_mw_ (**d**), T_conv_PEDOT (**e**), and T_mw_PEDOT (**f**) prepared samples.

To establish the textural characteristics, the nitrogen adsorption–desorption technique was applied. Thus, it was found that the synthesized composites preserved the TiO_2_ mesoporosity, exhibiting type IV isotherms (Figure 2), accompanied by an H2 hysteresis loop, which is characteristic of ink-bottle interconnected pores [26]. As well, the preserved mesoporosity was proved by DFT (Density Functional Theory) pore size distributions (Figure 2) derived from registered N_2_ sorption isotherms. Thus, the found pore diameters are in the range of 2.9–5.6 nm, each sample being characterized by 2 main pore sizes with maxima at approximately 3 nm and 5 nm, respectively. All results are listed in Table 1 and compared. Further, it has to be mentioned that in the case of the T_conv_PEDOT sample, a more advanced narrowing effect has been observed. Thus, only the T_conv_PEDOT sample shows a decrease in the pore size from 5.6 nm (of the T_conv_ sample) to 4.5 nm, meaning that this is the only case of a thin PEDOT film formation, of about 0.55 nm thickness, on the titania pore walls. The same trend is followed by the calculated pore volumes, which have been reduced with PEDOT deposition by 46% and 33% for T_conv_PEDOT and T_mw_PEDOT, respectively. This means that a thin uniform film of PEDOT forms on the TiO_2_ pore walls, in the case of the T_conv_PEDOT sample, while a complete filling of part of pores with PEDOT polymer occurs when the T_mw_ sample is used. Moreover, this finding is actually in good agreement with XRD results, which prove the formation of a bulky amorphous polymer phase. Further, by applying the BET theory to the registered isotherms, the specific surface areas were calculated, and they are listed in Table 1. As observed, the BET-specific surface areas of TiO_2_/PEDOT systems are still high, meaning that the PEDOT polymer did not agglomerate or overly collapse the TiO_2_ nanostructure, besides the fact that the simple PEDOT has a very poor porosity characteristic. Therefore, the specific surface areas of T_conv_PEDOT and T_mw_PEDOT have been reduced by only 33% and 36%, respectively.

#### 3.1.3. Morphological Characterization

**Figure 3:**
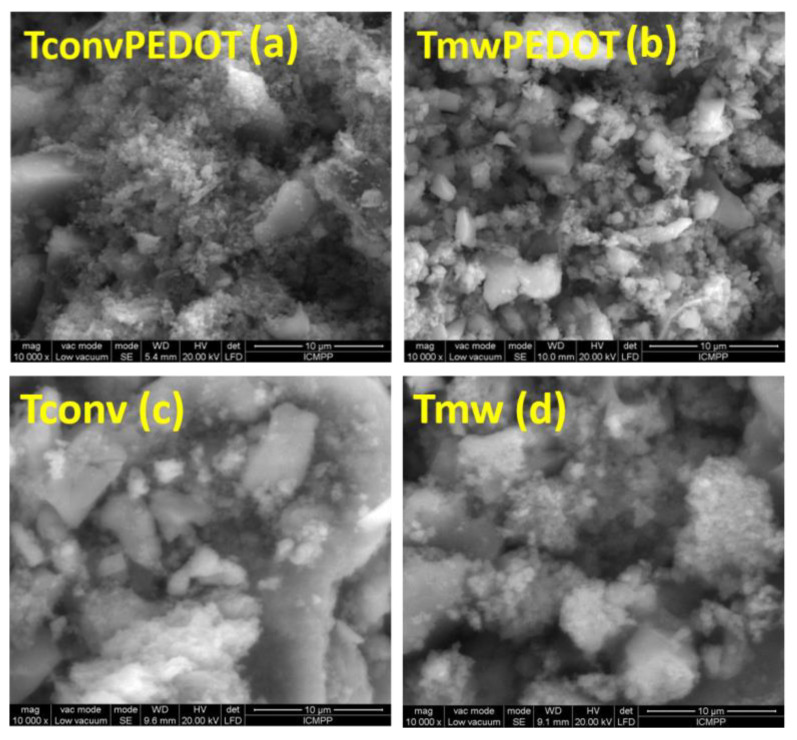
SEM images of the TiO_2_ and TiO_2_/PEDOT prepared samples under conventional and microwave-assisted synthesis: (**a**) conventionally thermally treated TiO_2_/PEDOT, (**b**) microwave treated TiO_2_/PEDOT, (**c**) conventionally thermally treated TiO_2_, (**d**) microwave-treated TiO_2_.

SEM microscopy was employed to investigate the morphology of the synthesized samples. The acquired SEM images are shown in Figure 3. A clear distinction between two different grain shapes of the TiO_2_/polymer systems can be seen in the SEM images, corresponding to TiO_2_ and PEDOT microstructures, respectively. Consequently, the TiO_2_ phase particles do not have an uniform shape but are characterized by well-defined edges, and these particles exhibit larger sizes compared to the spherical PEDOT particles, no matter the thermal treatment used during the synthesis procedure. It is proven that the EDOT polymerization process did not modify the titania morphology, with surface deposition of polymer particles being noticed.

#### 3.1.4. Investigation of the Surface Chemistry by FTIR Spectroscopy

**Figure 4:**
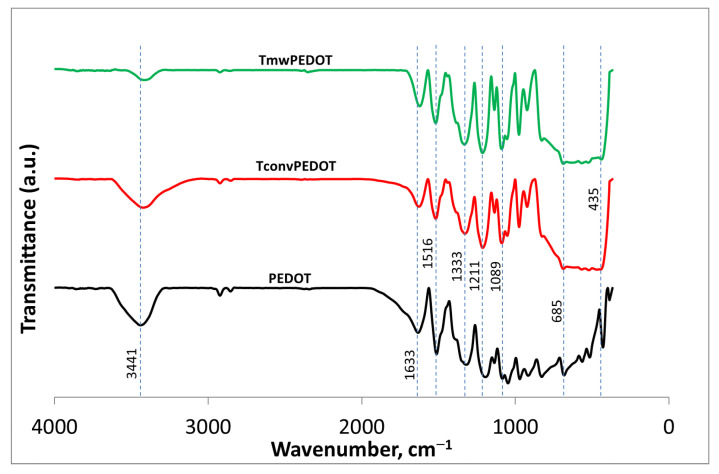
FT-IR spectra of PEDOT, TiO_2,_ and TiO_2_/PEDOT composites.

Fourier Transform Infrared Spectroscopy was employed to study the surface chemistry of the synthesized systems. FTIR spectra of all TiO_2_/PEDOT nanocomposites indicate characteristic bands for both PEDOT and TiO_2_ components. As observed in Figure 4, the characteristic band for the C–S stretching, centered at 685 cm^−1^ for the PEDOT sample [27], slightly shifts towards higher wavenumbers, 688.5 cm^−1^, for both prepared composites. The bands around 1211 and 1089 cm^−1^ correspond to the asymmetrical and symmetrical stretching of the C–O–C bonds that are characteristic of the ethylene-dioxy group, while the bands at 1514 and 1333 cm^−1^ are due to the C = C and C–C bonds that extend into the thiophene ring [27,28]. A higher shift of 6 cm^−1^ is noticed for the C = C stretching in the case of the TiO_2_/PEDOT. Furthermore, an additional band around 435 cm ^−1^, characteristic of titanium oxide bonds (Ti–O–Ti), is registered for the 2 samples containing metal oxide. The band centered at 1633 cm^−1^ and the broad band centered at 3441 cm^−1^ for the PEDOT sample confirm the presence of the hydroxyl groups on its surface (bending vibrations and stretching vibrations, respectively) [29,30]. For the nanocomposites, these bands are slightly shifted towards smaller wavenumber values.

#### 3.1.5. X-ray Photoelectron Spectroscopy

To gain information about the chemical structure at the surface of the synthesized samples, the XPS technique was considered. The survey spectrum of the nanocomposites revealed the presence of four elements: Ti (2p), O (1s), C (1s), and S (2p), which are the elemental markers for TiO_2_ and PEDOT phases, validating the successful synthesis of the TiO_2_/PEDOT nanocomposites. Table 2 shows the atomic semi-quantitative surface composition of all three samples, obtained by XPS analysis.

**Figure 5:**
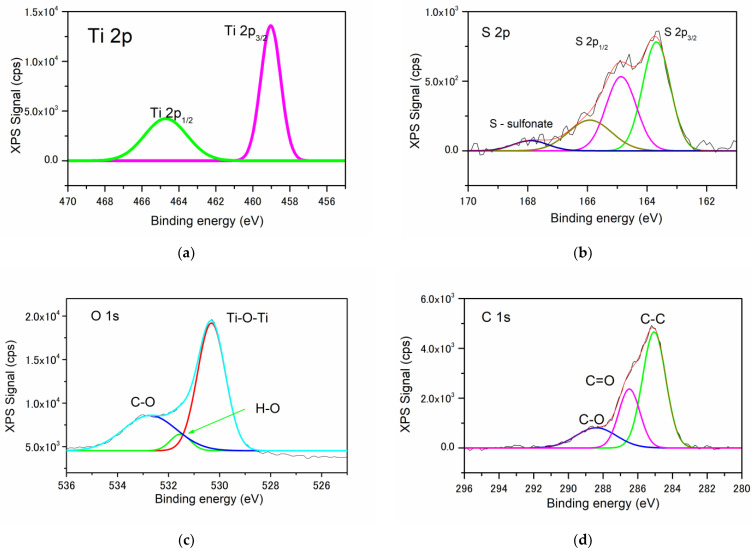
High-resolution XPS spectra of Ti 2p (**a**), S 2p (**b**), O 1s (**c**), and C 1s (**d**) of the T_conv_PEDOT sample.

Further, the high-resolution deconvoluted spectra of Ti2p, S2p, O1s, and C1s of the T_conv_PEDOT sample are shown in Figure 5a–d. All XPS peaks were deconvoluted using the Gaussian method. Thus, the Ti2p peak was deconvoluted into two main peaks positioned at 459.03 eV (Ti2p_3/2_) and 464.71 eV (Ti2p_1/2_), which correspond to Ti^4+^, characteristic of the TiO_2_ framework [31,32]. Further, the S2p peak was found to consist of 4 deconvoluted peaks, 2 of which are centered at 164.87 and 163.69 eV and assigned to spin-split doublets of S2p_1/2_ and S2p_3/2_, respectively [33]. Since a Ti–S bond was also reported in the range of 163–164 eV, a higher peak intensity is registered for S2p_3/2_ compared to that of S2p_1/2_. This may lead to the conclusion that a bond was formed between TiO_2_ and PEDOT polymer during the synthesis process [24]. The low-intensity peak centered at 168 eV could be associated with sulfonates [34], formed during the oxidative polymerization of PEDOT. The O1s deconvoluted spectra (Figure 5c) show 2 main peaks centered at 530.3 eV and 532.7 eV, which can be ascribed to Ti-O-Ti bonds in TiO_2_ [35] and C-O bonds in the thiophene ring of PEDOT. The small peak at 531.5 eV was reported by Matouk et al. [36] and Ghafourisaleh et al. [37] as hydroxide contamination, whereas Schultheiss et al. [34] attributed it to oxygen atoms from the sulfonate group, this being in good agreement with the results of the S2p analysis. The C1s spectra (Figure 5d) exhibit three peaks, which can be assigned to C–C/C–H, C–O-C, and C=O bonds of PEDOT [34]. These results constitute good evidence that a TiO_2_/PEDOT system was obtained.

#### 3.1.6. Optical Properties

**Figure 6:**
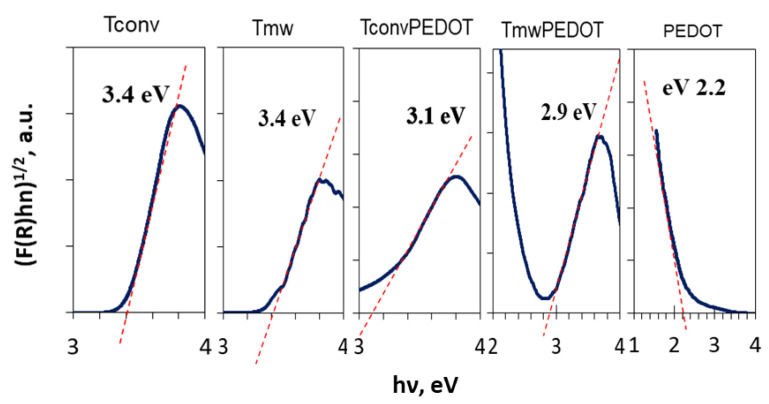
Tauc plots of the synthesized pure TiO_2_, PEDOT, and TiO_2_/PEDOT systems.

As the photocatalytic characteristic of a photocatalyst depends on light absorption, the band gap energy has to be determined. Thus, UV-DR spectra were registered and data were exploited to find out information on the optical band gap. Therefore, Figure 6 shows the Tauc plots obtained from diffuse reflectance spectra with Kubelka–Munk function for TiO_2_ and TiO_2_-PEDOT coated photocatalysts annealed by conventional (T_conv_ and T_conv_PEDOT) and microwave-assisted (T_mw_ and T_mw_PEDOT) heating methods. Band gap energy values thus determined are presented in Table 1. By analyzing the obtained values for the band gap energy, T_conv_ and T_mw_ photocatalysts were found to exhibit band gaps of 3.4 eV, which is favorable to the UV photocatalytic process, this being comparable with other studies presented in the literature reporting a value of 3.2 eV for commercial titanium oxide [1]. Moreover, as expected for PEDOT-containing samples, lower band gap values of 3.1 eV for the T_conv_PEDOT sample and 2.9 eV for the T_mw_PEDOT sample were found, confirming the hypothesis that composite photocatalysts incorporating PEDOT would exhibit enhanced response to visible light in comparison to bare TiO_2_. This is the result of heterojunction between two semiconductors, TiO_2_ and PEDOT, whose electronic band structures match well, thus enhancing the separation of photogenerated charge carriers. Similar results were obtained by Rahman (2020) when PANI (polyaniline), another conducting polymer, was used to sensitize TiO_2_ photocatalyst [38]. Therefore, the synthesized TiO_2_/PEDOT systems could be successfully used as photocatalysts for the decomposition of many harmful organic compounds under UV- and visible-light irradiation.

### 3.2. Photocatalytic Degradation of Congo Red Dye

The photocatalytic activity of a catalyst depends not only on the optical band gap but also on the number of active sites distributed on a large surface promoting the adsorption of organic molecules, thus increasing their degradation. In this regard, considering both optical characteristics and surface area, the photocatalytic efficiency of the prepared TiO_2_ and TiO_2_/PEDOT systems has been evaluated by the degradation of Congo Red dye. During the initial phase of the experiments, a blank test was conducted to assess if the solution could undergo decolorization solely through photolysis, without the presence of a catalyst. The findings indicated a slight reduction in the intensity of the absorption band of CR, with a degradation yield of merely 0.98% after 30 min of light exposure. In addition, adsorption experiments were conducted in the dark to examine the time-dependent dye adsorption behavior. It was observed that dye adsorption occurred within the first 5 to 10 min for all the samples under investigation. Thus, to achieve adsorption/desorption equilibrium, 30 min of adsorption in the dark preceded the turning on of the UV or visible lamp for all photocatalytic experiments.

#### 3.2.1. Effect of Catalyst Dose

**Figure 7:**
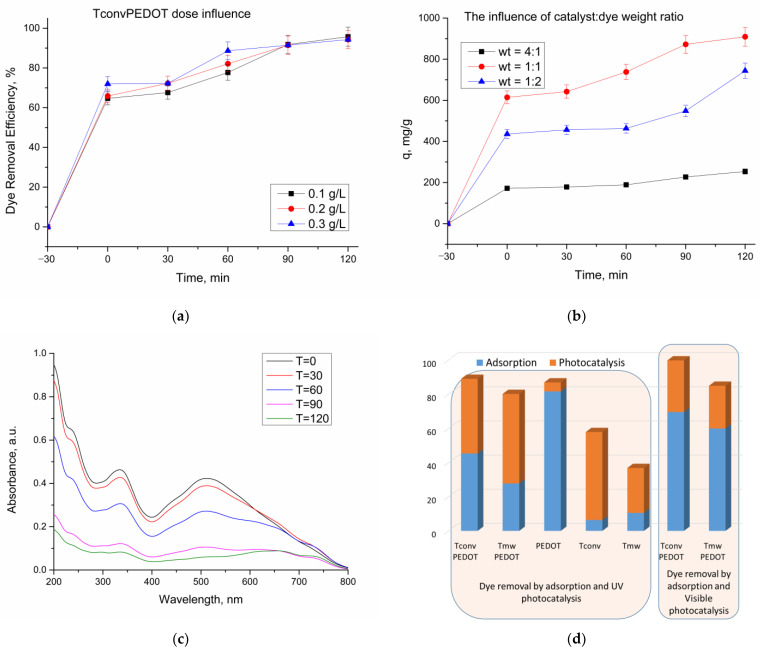
Photocatalytic degradation of Congo Red dye under various conditions: (**a**) effect of catalyst dose under UV exposure (catalyst: T_conv_PEDOT, 100 mg/L CR solution, 50 mL); (**b**) effect of catalyst:dye weight ratio under UV exposure (for 4:1 wt—0.01 g T_conv_PEDOT, 50 mg/L CR solution, 50 mL; for 1:1 wt—0.005 g T_conv_PEDOT, 100 mg/L CR solution, 50 mL; for 1:2 wt—0.005 g T_conv_PEDOT, 100 mg/L CR solution, 100 mL); (**c**) UV–Vis analysis of photocatalytic degradation of Congo Red dye (CR initial concentration = 100 mg/L, T_conv_PEDOT catalyst loading = 0.1 g); (**d**) dye removal through adsorption and photodegradation under UV and visible light.

In the second part of the photocatalytic experiments, the influence of catalyst dose on the photodegradation process of CR dye under UV light was investigated. For this purpose, different quantities of T_conv_PEDOT catalyst (0.1 g/L, 0.2 g/L, 0.3 g/L) were considered in the photodegradation experiments with CR dye aqueous solution (50 mL, C_0_ = 100 mg/L), and the obtained results are depicted in Figure 7a. It was noticed that when the dose increased from 0.1 g/L to 0.2 g/L or 0.3 g/L, the adsorption efficiency slightly increased from 64% to 65% and 71%, respectively. However, the total removal of the dye from the solution after 120 min of the photocatalytic reaction did been improve when a larger quantity of catalyst was added, the removal efficiencies being around 95% for all experiments. The conclusion drawn was that increasing the dose of the catalyst beyond 0.1 g/L, did not significantly improve the efficiency of dye removal from the solution. Therefore, this particular dosage was determined to be the optimum efficient dose for the T_conv_PEDOT catalyst.

#### 3.2.2. Determination of CR Dye Removal Capacity

Under the same photocatalytic conditions, the CR dye removal capacity of the prepared TiO_2_/PEDOT system was evaluated. Thus, Figure 7b shows the evolution of CR dye removal capacity (q), expressed as milligrams of CR removed from the solution per gram of catalyst, in the presence of the T_conv_PEDOT synthesized sample, with different catalyst:dye weight ratios. The weight ratios of 4:1, 1:1, and 1:2 were conducive to dye removals of 254 mg/g, 909 mg/g, and 743 mg/g, respectively, after 120 min of photocatalytic reaction. It was concluded that the most favorable catalyst:dye weight ratio for CR dye removal was 1:1, when a quantity of 0.005 g T_conv_PEDOT was employed for the degradation of 50 mL CR solution, C_0_ = 100 mg/L. The evolution of UV–Vis spectra of CR solution (100 mg/L CR solution, 50 mL) during its photocatalytic degradation in the presence of T_conv_PEDOT catalyst is shown in Figure 7c. It is observed that the characteristic peaks of CR dye show an absorbance decrease over time, suggesting the photodegradation of CR.

Using the found optimum values for the photocatalytic process parameters, the dye degradation efficiencies of the synthesized samples are presented in Figure 7d. One notable result is the high adsorption capacity found for CR dye on a PEDOT sample in the dark, reaching a removal efficiency of 81%. In contrast, the bare TiO_2_ samples exhibit low adsorption capacity compared to PEDOT conjugated polymer. Further, by PEDOT insertion through the TiO_2_ matrix, an increase in adsorption capacity could be identified, a finding that is advantageous for concentrating the CR molecules around photocatalytic sites on the TiO_2_ surface. Thus, it is worth mentioning that in the prepared TiO_2_/PEDOT systems, a synergistic effect of both components is observed. Accordingly, it was found that the overall efficiency of the TiO_2_ samples and TiO_2_/PEDOT systems followed the order T_conv_PEDOT > T_mw_PEDOT > T_conv_ > T_mw_. When considering only the photocatalytic activity, the most efficient TiO_2_/PEDOT system was the one that contains microwave-derived TiO_2_ (T_mw_PEDOT), highlighting the significant influence of the synthesis route on determining the properties of the catalytic material. In comparison to the samples that involved conventional thermal-treatment-assisted synthesis, both T_mw_ and T_mw_PEDOT samples demonstrate lower surface areas and mean pore diameters, as indicated in Table 1. This observation suggests that these characteristics play a critical role in the photocatalytic process as well. Moreover, these results are in good agreement with the ones obtained from the Tauc plots, with the T_mw_PEDOT sample exhibiting the lowest band of only 2.5 eV, which indicates the visible-light activation of the photocatalyst. This is further shown in Figure 7c and Table 3, where both dye concentration and TOC were measured after the visible-light photodegradation process and confirmed the improvement of the photocatalytic activity and its enhancements regarding visible-light harvesting.

Moreover, the main conclusion that could be drawn focuses on the enhanced photocatalytic activity of the prepared composite systems, which is owed to the synergistic effect of two components, as well as the tailored conduction band of the two semiconductors, which enables the transfer of charge carriers across PEDOT-O-Ti heterojunctions under light illumination [3]. Very few studies report the use of TiO_2_/PEDOT systems as photocatalysts. However, Katančić [3] reported the removal of Bisphenol A using 1.1 g/L TiO_2_-PEDOT photocatalyst activated by simulated solar light, with 60% efficiency, in 60 min.

In conclusion, the synthesized novel TiO_2_/PEDOT systems demonstrate great potential as cost-effective photocatalysts for application in water treatment and environmental remediation. Remarkably, these materials exhibit exceptional degradation capabilities towards Congo Red dye, achieving removal efficiencies of up to 90% when exposed to UV light and 99% when utilizing visible light, even with a small catalyst dosage of 0.1 g/L and a 2 h degradation process. Their performance is comparable with that of other photocatalysts reported in the literature. Furthermore, a greater efficiency under visible-light irradiation has been obtained, as could be observed in Table 4.

#### 3.2.3. Identification of Reactive Species Responsible for CR Photo-Oxidation

**Figure 8:**
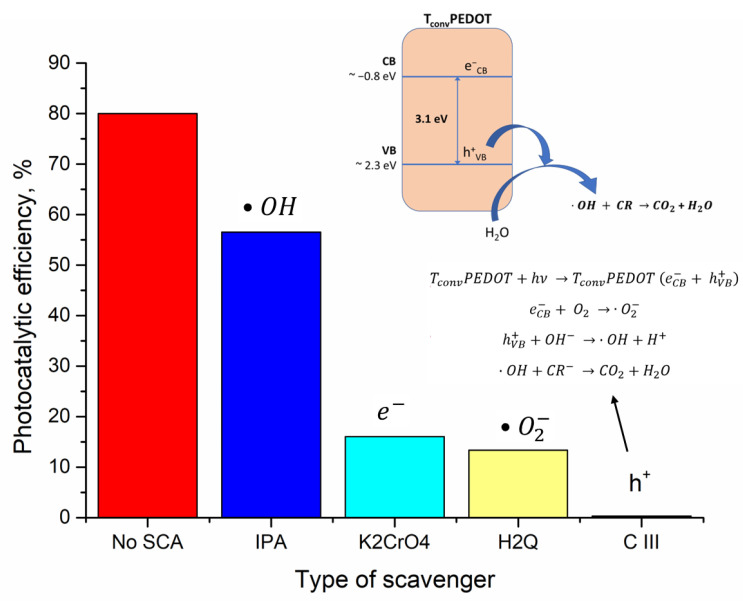
Photocatalytic tests in the presence of scavengers (0.005 g T_conv_PEDOT, 50 mL CR solution, C_0_ = 100 mg/L).

Aiming to elucidate the mechanism of degradation of CR dye by TiO_2_/PEDOT prepared systems, different scavengers such as IPA, 10 mmol L^−1^ (scavenger for •OH radical); potassium chromate, 25 mmol L^−1^ (scavenger for e^−^) [41]; H_2_Q, 0.25 mmol L^−1^ (scavenger for •O_2_^−^) [42]; and CIII, 10 mmol L^−1^ (for h^+^ traping) [43] were used. This allowed us to investigate the role of active species involved in the photocatalytic degradation of CR dye over the synthesized TiO_2_/PEDOT system. Hence, radical trapping control tests of the T_conv_PEDOT sample to study the oxidation of CR under UV light were carried out. Likely, most of the photocatalytic processes are usually conducted by free •OH radicals. Thus, to test the formation of •OH radicals in the photocatalytic mixture, isopropyl alcohol was used. Thus, as seen in Figure 8, the photo-oxidation rate of CR remained at 56.5% when IPA was used as •OH radicals scavenger, being lower than that of a blank no-scavenger test. This assumes that •OH radicals do not play the main role in the photo-oxidation of CR dye. However, when K_2_CrO_4_ and H_2_Q were used as scavengers for e^−^ and •O_2_^−^, the photodegradation rate of CR dye over T_conv_PEDOT photocatalyst was reduced to 16% and 13.3%, respectively. These results suggest moderate participation of e^−^ and •O_2_^−^ reactive species in the photocatalytic degradation of CR dye in the presence of T_conv_PEDOT photocatalyst, under UV-light irradiation. Furthermore, the photocatalytic activity of the T_conv_PEDOT system decreased considerably to 0.03% when CIII was added as a quencher. This finding demonstrates that h^+^ plays the most important role in the photo-oxidation activity of synthesized T_conv_PEDOT photocatalyst. Thus, the holes (h^+^) are considered the primary oxidizing species in photocatalytic degradation of CR dye over the T_conv_PEDOT photocatalyst prepared, as related to other TiO_2_-based photocatalysts reported in the literature elsewhere [44]. In such a case, the photogenerated h^+^ can carry out the direct oxidation of CR dye molecules, while the latter could act as electron donors [45].

## 4. Conclusions

In the present study, novel TiO_2_ and TiO_2_/PEDOT catalysts were successfully synthesized using the US-assisted sol–gel method. Both conventional heat annealing and a microwave irradiation technique were employed to eliminate the surfactant leaving behind voids and create porous structures. Characterization techniques proved the successful formation of TiO_2_/PEDOT nanocomposites. The features of the synthesized powder samples were examined to evaluate their effectiveness in degrading model pollutant molecules, specifically Congo Red dye. It was found that the heterostructuring of TiO_2_ with PEDOT polymer resulted in lower band gap materials, which not only could be activated by UV light but also are highly efficient under visible-light irradiation. It was proven that both microwave and conventional thermal treatment led to the synthesis of highly efficient new photocatalysts that are fitted to be used in the most economical and energy-sustainable wastewater treatment, by using solar energy. Thus, the high dye decolorization (of 85–99%) and dye mineralization (of 60–70%) degrees were achieved when using only 0.1 g/L (T_mw_PEDOT and T_conv_PEDOT) of the synthesized catalysts. Further, it was found that holes (h^+^), formed at the TiO_2_/PEDOT interface, are the main reactive species responsible for the photo-oxidation of CR dye. Moreover, the increased performance seems to be related to the inhibition of the electron–hole recombination, and the PEDOT-conjugated-polymer-modified TiO_2_ porous photocatalysts are expected to demonstrate a broad range of utility for other catalytic reactions.

## Figures and Tables

**Table 1:** Structural and textural parameters characterizing the prepared materials and their photocatalytic efficiencies under UV and Vis lights.

Sample	FWHM (deg)	D_101_ (nm)	d_101_ (Å)	a_0_ (Å)	D_WH_ (nm)	Ε (*10^−2^)	Eg, (eV)	S_BET_, (m^2^/g)	V_tot_, (cm^3^/g)	d_pore_ (nm)	E (%) UV Photocatalysis	E (%) Vis Photocatalysis
T_conv_	1.469	5.48	3.516	4.06	10.71	1.03	3.4	129	0.24	5.6 3.2	37	-
T_mw_	0.920	8.75	3.500	4.04	11.92	0.96	3.4	215	0.27	4.7 2.9	58	-
PEDOT	5.122	1.57	3.481	4.02	-		-	12	0.05	-	86	-
T_conv_PEDOT	1.588	5.07	3.514	4.06	9.33	0.78	3.1	87	0.13	4.5 3.1	90	99
T_mw_PEDOT	2.44	3.3	3.537	4.084	5.19	0.0036	2.5	137	0.18	4.6 2.9	80	85

FWHM—Full Width at Half Maximum for the representative peaks; D_101_—crystallite size determined using the Scherrer formula; d_101_—interplanar spacing; a_0_—unit cell parameter; D_WH_—crystallite size calculated using the WH theory; ε—lattice strain; Eg—band gap energy; S_BET_—specific surface area calculated by applying Brunauer–Emmett–Teller theory; V_tot_—total pore volume measured at P/P0 = 0.95 (cm^3^/g); d_pore_—mean pore diameter calculated with DFT method, nm.

**Table 2:** XPS atomic surface chemical composition (%).

Sample	C 1s	O 1s	S 2p	Ti 2p3
PEDOT	71.3	21.8	6.9	-
T_conv_PEDOT	39.0	41.6	2.4	17.1
T_mw_PEDOT	38.9	41.3	2.1	17.7

**Table 3:** Mineralization of CR over synthesized TiO_2_/PEDOT systems, estimated by determining TOC removal, under both UV and visible-light irradiation.

Sample	Mineralization (%)	
	UV Light	Visible Light
T_conv_PEDOT	59.32	52.32
T_mw_PEDOT	43.74	39.34

**Table 4:** A comparison of the CR photocatalytic degradation performance of different photocatalysts reported under UV and visible-light irradiation.

Sample	Light Source	CR Solution	Sample, mg	Time of Exposure (min)	Photo-Decolorization (%)	Reference
TiO_2_-C,N	Visible	100 mL 10 mg/L	50 mg	45	91	[39]
	UV	100 mL 10 mg/L	50 mg	45	98	
Al-SrTiO_3_	Visible	100 mL 10 mg/L	20 mg	90	68	[40]
	UV	100 mL 10 mg/L	20 mg	90	81	
T_conv_PEDOT	Visible	50 mL 100 mg/L	5 mg	120	99	This work
	UV	50 mL 100 mg/L	5 mg	120	90	

## Data Availability

Not applicable.
